# Hepatitis C Micro‐Elimination in a Deprived Coastal Town: Lessons From Blackpool

**DOI:** 10.1002/puh2.70344

**Published:** 2026-08-10

**Authors:** Sarah E. Blagden, Emma L. MacFarlane, Dianne C. Draper

**Affiliations:** ^1^ Blackpool Researching Together (NIHR Blackpool Health Determinants Research Collaboration) Blackpool UK; ^2^ Public Health Directorate Blackpool UK; ^3^ Division of Health Research Lancaster University Lancaster UK; ^4^ Delphi Medical Limited Blackpool UK

**Keywords:** evaluation, hepatitis C, micro‐elimination, people who inject drugs

## Abstract

**Background:**

Blackpool is the most deprived local authority district in England and consistently has one of the highest rates of hepatitis C virus (HCV) nationally. This study describes a multi‐stakeholder approach to address low levels of HCV testing within drug and alcohol treatment services in Blackpool and evaluates the outcomes of this.

**Methods:**

This study is a rapid evaluation utilising the UK Health Security Agency's rapid evaluation model. Individual‐level data were extracted from adult drug and alcohol treatment services in Blackpool for all clients accessing services between April 2024 and July 2025. Analysis consisted of descriptive statistics to determine testing and treatment outcomes against national HCV micro‐elimination criteria and to describe the client population. Hypothesis testing was carried out to investigate differences between clients testing positive and negative, and binary logistic regression to identify factors associated with a positive HCV test.

**Results:**

Of 1294 clients attending services, 1071 had a valid HCV test result recorded during the period. Of these, 32 tested positive for active HCV infection (3%), >90% of whom initiated treatment. There was significantly greater heroin use, crack use and injecting behaviour amongst those testing positive. Injecting mediated the relationship with substance type and was the only factor independently associated with HCV infection.

**Conclusions:**

All micro‐elimination criteria were met, and drug and alcohol services in Blackpool achieved micro‐elimination. Moving forwards, services must sustain the culture changes that have embedded HCV testing within standard practice.

## Graphical abstract

By making hepatitis C virus (HCV) testing a routine part of care, Blackpool's drug and alcohol services dramatically improved HCV diagnosis and treatment and achieved HCV micro‐elimination. Continued commitment to this new way of working will be essential to sustain these gains.

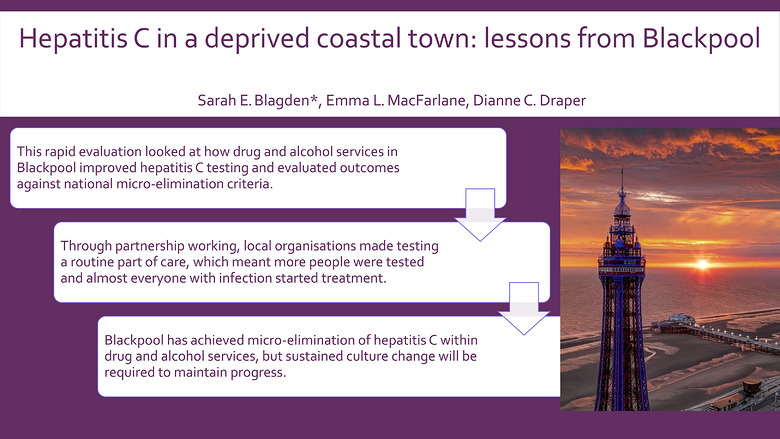

## 1 Introduction

### 1.1 What Was the Problem You Were Trying to Address?

Hepatitis C virus (HCV) remains a major global health concern, with an estimated 50 million people living with HCV worldwide and 240,000 people dying from HCV in 2022, primarily due to cirrhosis and liver cancer [1]. The World Health Organisation has set ambitious targets to eliminate HCV as a public health threat by 2030, aiming for a 65% reduction in mortality and a 90% reduction in new infections [2]. In the United Kingdom, considerable progress has been made. Between 2015 and 2023, the number of people living with chronic hepatitis C in England fell by 56.7%, now estimated at around 55,900 [3]. This decline is largely attributed to the rollout of highly effective direct‐acting antivirals (DAAs), which offer short, all‐oral treatment courses with cure rates exceeding 90% [2]. Despite these advances, challenges remain. This is particularly amongst people who inject drugs (PWID), where prevalence remains high, many individuals are undiagnosed, and reinfections occur [3].

Hepatitis C micro‐elimination is a focused public health strategy and offers a complementary approach to HCV elimination by targeting specific populations, services or geographic areas in which prevention, screening and treatment can be implemented more effectively through healthcare setting‐based interventions [3, 4, 5]. Within drug and alcohol services in England, micro‐elimination is defined as [3, 6]

- 100% of clients in structured treatment have been offered an HCV test.
- 100% of clients in structured treatment with a history of injecting have a valid HCV test (ever), with the result recorded.
- 90% of clients in structured treatment who have a history of injecting have had an HCV test in the last 12 months, with the result recorded.
- 90% of clients in structured treatment with a positive HCV (RNA) test result have initiated treatment.

This approach allows for faster, more tailored interventions and has led to successful outcomes in several UK services.

### 1.2 What Caused the Problem?

Blackpool is the most deprived local authority district in England and has significant health inequalities. The town has particularly high levels of drug use, with the highest rate of drug‐related deaths in the country [7]. As a result, Blackpool has consistently had one of the highest rates of hepatitis C in England, with a detection rate of 40.0/100,000 in 2022, compared to the England average of 25.1/100,000 [8]. Specialist drug and alcohol treatment services in England are commissioned by local authorities, and, at the time of writing, adult services in Blackpool are provided under the umbrella of ‘Horizon’. This is a partnership between Delphi Medical Limited, together with Renaissance UK and Blackpool Council [7]. In early 2024, Blackpool was identified by NHS England as an area with extremely low levels of HCV testing within its drug and alcohol treatment services. In recognition, local partners set out a multi‐agency plan to address this and to attempt to achieve micro‐elimination.

In this article, we describe the approach and interventions used in Blackpool to address the high prevalence of hepatitis C and low levels of testing within drug and alcohol services. We then evaluate the effectiveness of these interventions against micro‐elimination criteria.

## 2 Methods

This article, and the evaluation of Blackpool's approach to hepatitis C micro‐elimination, is structured using the nine questions of the UK Health Security Agency (UKHSA) rapid evaluation model:

1. What was the problem you were trying to address?
2. What caused the problem?
3. Describe the intervention.
4. What were the reasons for choosing this particular intervention?
5. What have you measured to demonstrate the initial problem has been addressed?
6. What were the outcomes?
7. Was the intervention delivered as planned?
8. What lessons have been learnt?
9. What next?

Application of this model is recommended by UKHSA to evaluate hepatitis C interventions [9]. Evaluation refers to activity that assesses the impact of an intervention or service delivery mode.

### 2.1 ‘Describe the Intervention’ and ‘What Were the Reasons for Choosing this Particular Intervention?’

In April 2024, drug and alcohol services in Blackpool commenced a journey to address low levels of HCV testing. This was carried out in partnership, with key stakeholders encompassing: Blackpool Council's Public Health team, Delphi Medical, Renaissance UK, Acorn Recovery Projects and the Specialist Liver Service at Blackpool Victoria Hospital. The key components of this approach, along with the rationale for their inclusion, are shown in Table 1.

**TABLE 1:** Components of the Blackpool hepatitis C micro‐elimination approach.

Component	Detail	Why was this component included?
**Data and IT systems**	During the project, Delphi was invited to join Gilead Sciences’ Provider Forum, which provided access to live data dashboards to actively monitor client testing and outcomes Computer systems were upgraded to enable Delphi to have timely and accurate access to test results The Lancashire and South Cumbria Operational Delivery Network (ODN) undertook a data deep‐dive to understand current testing levels	As a small geography and small, independent provider, Blackpool had previously lacked access to Gilead Sciences’ Provider Forum Services had struggled to record, track and visualise testing progress and outcomes, and HCV testing data were limited and difficult to access Computer systems were not delivering timely results, and services could be waiting several weeks for results, with some not received at all
**Internal monitoring, improvement and training**	Within Delphi, regular internal staff meetings were held to discuss further training needs, confidence and accountability for testing Comprehensive training was delivered to staff around HCV testing and counselling Incentive prizes were given to staff for the number of tests carried out Regular audits on testing progress were conducted	Hepatitis C was not seen as a priority when clients were entering the service due to other more pressing issues There was a lack of confidence amongst staff around HCV testing and counselling
**Partnership working**	Fortnightly meetings were implemented between treatment services and Blackpool Council's Public Health team Regular meetings with the Lancashire and South Cumbria ODN were implemented The pathway from drug and alcohol services into the Specialist Liver Service at Blackpool Victoria Hospital was reviewed and strengthened	As the commissioner of drug and alcohol services, Blackpool Council provided oversight and support to the micro‐elimination process Regular meetings enabled shared learning from successful testing initiatives in other areas
**Strengthened sampling pathways**	A central sample post box was installed in drug and alcohol services, and a protocol was implemented for peer checking of samples and labels	Previously, the laboratory would send incorrect labels due to confusion around the location of clients Samples would also be regularly rejected due to incorrect labelling
**Engagement with service users**	Supported by the hepatitis C trust, a sustained programme of initiatives was carried out to promote testing and engage service users, for example, Christmas sock exchange, present pack and treats, Halloween party and vouchers Clients requiring HCV treatment were supported by outreach nurses from Delphi and the hepatitis C trust	Previously, services had struggled to engage some service users to participate in testing, and particularly when treatment was required

### 2.2 What Have You Measured to Demonstrate the Initial Problem Has Been Addressed (Analysis Plan)?

All analyses were conducted using IBM Statistics Version 31. Summary HCV testing data were extracted by Delphi Medical for all clients accessing adult drug and alcohol services in Blackpool between April 2024 and July 2025. This was utilised to generate a cascade of care to visually depict testing and treatment outcomes against micro‐elimination criteria.

In addition, pseudo‐anonymised individual‐level data were extracted for clients with a valid HCV test result recorded during this period. For these clients, data were extracted on HCV test outcome as well as key demographic characteristics (age, ethnicity, sex, injecting status and substances used). For those clients who tested positive, additional data were extracted on sexuality, age at first drug use, co‐morbidities and presence of disability. After data cleaning, descriptive statistics were calculated and tabulated for the entire cohort, those testing positive and those testing negative. Where cell counts were <5, these were suppressed to prevent re‐identification of individuals, with secondary suppression required in other cells in some instances. Hypothesis testing was carried out to identify statistically significant differences between the cohorts testing positive and negative. Next, univariable logistic regression was carried out to explore the association between independent variables (demographics) and the dependent variable (hepatitis C test outcome). Univariable associations were statistically significant if *p* ≤ 0.05. Finally, multiple logistic regression was used to investigate the effect of independent variables, adjusted for the effect of confounders. Age and sex were entered into the model a‐priori, with further variables entered if, upon univariable regression analysis, the p value was ≤0.2. Within all analyses, PWID were defined as people with a history of ever injecting drugs, as described in other studies [10].

## 3 Results

### 3.1 What Were the Outcomes?

#### 3.1.1 Key Outcomes

**FIGURE 1:**
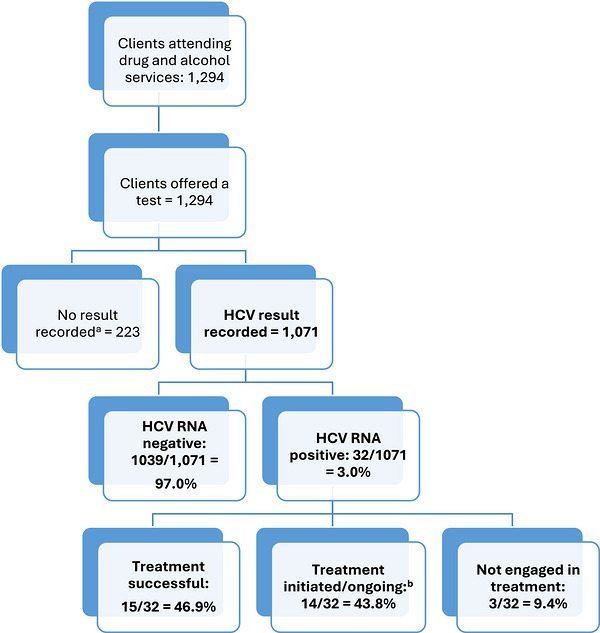
Outcomes of hepatitis C virus testing for all adult clients tested in Blackpool drug and alcohol services (April 2024–July 2025). ^a^Includes: refusal of testing, sampling/laboratory issues and known HCV cases. ^b^Incorporates all individuals where treatment had been initiated (including those awaiting testing for response to treatment, undergoing re‐treatment due to first‐line treatment failure and unrelated death after treatment commencement and treatment out‐of‐area). ^c^As of June 2025. **Progress against hepatitis C micro‐elimination criteria for drug and alcohol treatment services for cohort tested**: 100% of people who access the service have been offered a test = **100%** 100% of people with a history of injecting have been tested (ever), with a result recorded = **100%** 90% of people in structured treatment with a history of injecting drugs who are at risk have had a test in the last 12 months, with the result recorded = **90.6%** ^c^ Treatment has been initiated for 90% of people who test (HCV RNA) positive = **90.6%**.

During the period analysed (April 2024–July 2025), there were 1294 clients attending adult drug and alcohol treatment services in Blackpool. Of these, 1065 (82.3%) either did not have a valid HCV test result recorded ever (non‐injecting clients) or a valid result from the previous 12 months (injecting clients). All clients were offered HCV testing during the period of interest, and Figure 1 displays the outcomes of testing, including against hepatitis C micro‐elimination criteria for drug and alcohol services.

As shown in Figure 1, 100% of clients were offered a test during the period of interest. Across the cohort, results were recorded for 1071 individuals. Of these, 32 (3.0%) tested positive for active (RNA‐positive) HCV infection that had previously been undiagnosed. Of those testing positive, 15 (46.9%) had successfully completed treatment with sustained virological response (SVR). A further 14 (43.8%) had initiated treatment and were at varying stages of treatment, including those awaiting testing for response to treatment and re‐treatment due to first‐line treatment failure. Combined, this meant that treatment had been initiated for 90.6% of people testing positive.

#### 3.1.2 Client Characteristics

**FIGURE 2:**
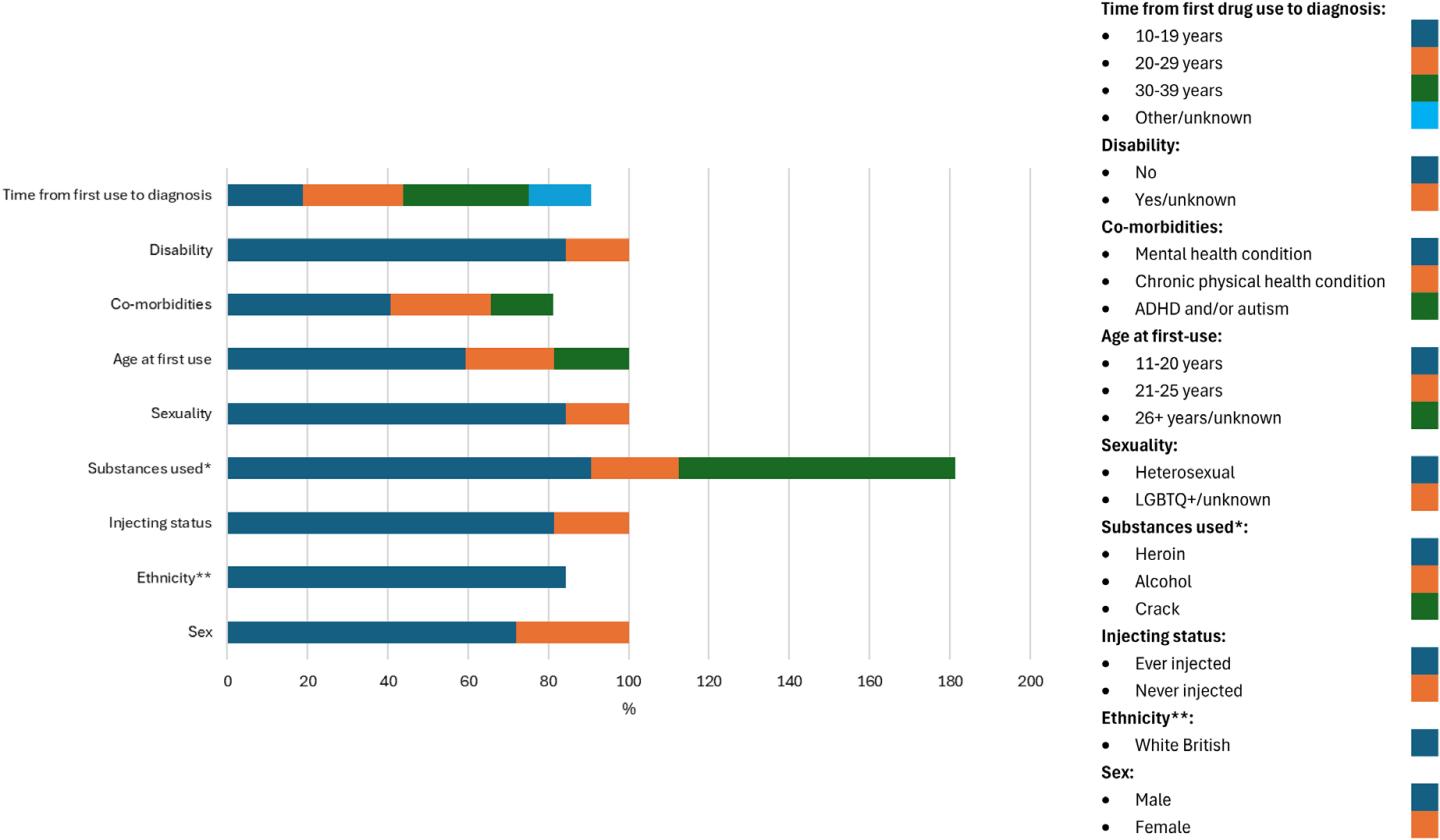
Characteristics of clients testing positive for hepatitis C in Blackpool drug and alcohol services: April 2024–July 2025. *Total is >100 due to individuals using multiple substances. **Count <5 (<15.6%) from other ethnic groups; therefore, exact totals have been suppressed.

Table 2 summarises the characteristics of all clients, along with breakdown for those testing positive and negative. Figure 2 provides additional information about those clients testing positive.

**TABLE 2:** Characteristics of all clients tested for hepatitis C virus (HCV) in Blackpool drug and alcohol services presented by all clients, those testing positive and those testing negative—April 2024–July 2025.

	All cases (*n* = 1071)	HCV‐positive test (*n* = 32)	HCV‐negative test (*n* = 1039)	
Continuous variable	Mean (standard deviation—SD)	Mean (SD)	Mean (SD)	*p* value
Age—years	47.0 (9.4)	45.9 (8.4)	47.0 (9.4)	0.504^a^
**Categorical variable**	***N* (%)**	***N* (%)**	***N* (%)**	***p* value**
Sex:				
Male Female	688 (64.2)	23 (71.9)	665 (64.3)	0.379^b^
	378 (35.3)	9 (28.1)	369 (35.7)	
Ethnicity:				
White British Other Missing	985 (92.0)	>27 (>84.4)	954 (91.8)	0.300^b^
	7 (0.7)	<5 (<15.6)	6 (0.6)	0.192^c^
	79 (7.4)	0	79 (7.6)	0.163^c^
Injecting status:				*<0* *.001* ^b^
Never/Unknown People who inject drugs	589 (52.1)	6 (18.9)	553 (53.2)	
	482 (45.0)	26 (81.3)	456 (43.9)	
Substances used:				
Heroin	621 (58.0)	29 (90.6)	592 (57.0)	*<0.001* ^b^
Alcohol	398 (37.2)	7 (21.9)	391 (37.6)	0.069^b^
Crack	427 (39.9)	22 (68.8)	405 (39.0)	*<0.001* ^b^
Cocaine	84 (7.8)	<5 (<15.6)	81 (7.8)	0.743^b^
Cannabis	148 (13.8)	<5 (<15.6)	144 (13.9)	0.826^b^
Benzodiazepines	77 (7.2)	<5 (<15.6)	75 (7.2)	0.835^b^
Synthetic cannabinoid receptor agonists	6 (0.6)	<5 (<15.6)	5 (0.5)	*0.048* ^b^
Ketamine	10 (0.9)	0	10 (1.0)	0.577^b^
Amphetamines	26 (2.4)	0	26 (2.4)	0.365^b^
Other opiates	73 (6.8)	0	73 (7.0)	0.120^b^

*Note:* Italics denote statistically significant *p*‐values.

^a^Independent samples *t*‐test.

^b^Chi‐square test.

^c^Fisher's exact test.

As shown in Table 2, the median age of clients tested was 47 years, with a majority (64.2%) of these being male, and no statistically significant differences in age or gender between those testing positive or negative. In keeping with the population of Blackpool, the cohort tested were almost exclusively White British. Across all clients, 45% had a history of injecting drugs, and, unsurprisingly, this was significantly higher amongst those who tested positive for HCV (81.3%, *p* < 0.001). In terms of the substances used, the most prevalent across all three categories were heroin, alcohol, crack, cocaine, cannabis and benzodiazepines. Amongst those testing positive, there was significantly greater heroin (90.6% vs. 57%, *p* < 0.001) and crack (68.8% vs. 39.0%, *p* < 0.001) use. In contrast, other substances, such as ketamine, amphetamines and other opiates, did not feature amongst those testing positive for HCV.

As displayed in Figure 2, additional information was made available for clients who tested positive for HCV, which provided interesting insights into this cohort. This group had started using drugs at a young age, with 81.3% having confirmed drug use by age 25 years and 59.4% by age 20 years. Linked to this, time from first drug use to HCV diagnosis was very long, with 27.7 years the average duration. There was high prevalence of co‐morbidities, in particular mental health conditions, ADHD and autism.

#### 3.1.3 Associations With HCV‐Positive Result

As shown in Table 3, although there were univariable associations between PWID, heroin use and crack use, the only variable independently associated with HCV infection was ever injecting. This increased the odds of HCV infection almost three‐fold (OR = 2.951, 95% CI = 1.067–8.165).

**TABLE 3:** Results of univariate and multivariate analysis between patient characteristics and hepatitis C virus (HCV) infection.

	Univariate analysis				Multivariate analysis			
Independent variable	Odds ratio (OR)	95% confidence interval (CI) lower	95% CI upper	*p* value	OR	95% CI lower	95% CI upper	*p* value
Age	1.013	0.976	1.051	0.503	1.033	0.988	1.081	0.157
Female gender	0.705	0.323	1.540	0.381	0.614	0.273	1.382	0.239
White British ethnicity	0.195	0.023	1.669	0.328	—	—	—	—
People who inject drugs	5.540	2.261	13.574	*<0.001*	2.951	1.067	8.165	*0.037*
Substances used:								
Heroin	7.299	2.209	24.113	*<0.001*	4.233	0.944	18.993	0.060
Alcohol	2.155	0.923	5.029	*0.076*	0.628	0.240	1.644	0.344
Crack	3.444	1.614	7.348	*<0.001*	1.668	0.772	3.852	0.231
Cocaine	1.223	0.3665	4.103	0.744	—	—	—	—
Cannabis	0.826	0.307	2.569	0.826	—	—	—	—
Benzodiazepines	0.857	0.201	3.655	0.835	—	—	—	—
Synthetic cannabinoid receptor agonists	0.150	0.017	1.322	*0.087*	0.268	0.027	2.667	0.261

*Note:* Italics denote statistically significant *p*‐values.

## 4 Discussion

### 4.1 Was the Intervention Delivered as Planned?

During the period of interest, all elements of the intervention (as described in Table 1) were delivered successfully, and the four micro‐elimination criteria were met. As such, HCV micro‐elimination was achieved in adult drug and alcohol services in Blackpool. Given the large number of people requiring testing at the project's onset alongside the levels of reported drug use in an extremely deprived population, this represents a considerable achievement.

A small but significant minority of people who had not previously been identified tested positive for HCV, demonstrating that HCV is circulating amongst drug and alcohol service users in Blackpool. Of those testing positive, over 90% were being treated with DAAs or had achieved SVR. Historically, people who use drugs have been viewed as ‘hard to reach’ by health services due to factors such as drug use being illegal/stigmatised (making individuals reluctant to seek out or accept help), lack of a permanent address and inflexible appointments [11]. Our study adds to a growing body of evidence that this group can be effectively targeted, tested and treated.

Micro‐elimination approaches are multi‐faceted, multi‐stakeholder approaches, and it is difficult to pull out specific components that were/were not effective [11]. Several behavioural, structural and governance mechanisms likely contributed to the intervention's effectiveness. It is probable that embedding HCV testing within routine contacts will have reduced the cognitive and practical burden on clients, capitalising on established relationships and minimising the need to navigate unfamiliar healthcare environments [12]. As reported in other studies, the structural co‐location of testing, referral and treatment pathways reduced fragmentation and improved continuity of care, addressing local barriers such as poor integration with specialist liver services [12]. Governance factors also played a key role in terms of clear leadership from the Public Health team, shared objectives across organisations and real‐time communication between stakeholders. These features align with wider evidence showing that micro‐elimination succeeds when interventions are tailored to local systems, supported by strong inter‐agency governance and designed to reduce behavioural friction for high‐risk populations [11, 12, 13, 14].

### 4.2 What Lessons Have Been Learnt?

This project showcases the success of adopting care pathways for HCV diagnosis and treatment around facilities and services that people who use drugs are already accessing. It adds to a growing body of evidence that devolved care pathways are capable of HCV micro‐elimination in settings where HCV transmission is characterised by PWID [11, 13]. Decentralised care pathways, particularly in drug service settings, are key to increasing diagnosis of HCV, as shown by services that were early to achieve micro‐elimination; for example, NHS Tayside in Scotland, where the proportionate prevalence of HCV detected in drug services was substantially higher than in more traditional pathways within primary and secondary care [13]. Previous work has proven that adding pathways within settings such as drug treatment centres, prisons and community pharmacies sequentially increased the number of different individuals tested and cases identified with each pathway, suggesting that diverging populations are reached [13].

An interesting finding in this study was the very long time between first drug use and diagnosis, with clients testing positive in Blackpool, on average, much older (45.9 years) than reported elsewhere, for example, 34 years in a Scottish study [10]. It may be that individuals in Blackpool became infected at an older age; however, there is also the potential for infections to have been undetected for many years. This, alongside co‐existent alcohol use, suggests that there is the potential for significant liver damage amongst individuals testing positive in Blackpool. A previous French study of micro‐elimination in drug and alcohol services showed that a large number had severe liver scarring or cirrhosis at diagnosis [14].

### 4.3 What Next?

Although the focus and push to achieve micro‐elimination in Blackpool in 2024/2025 was extremely successful and produced good outcomes, it does not represent a single exercise. Though the backlog of never‐tested individuals in Blackpool has been addressed, the ongoing maintenance of micro‐elimination in Blackpool will depend upon sustained culture change within drug and alcohol services to make HCV testing, and the associated actions required to facilitate treatment, part of standard practice, rather than a one‐off event.

Our comparative analysis of individuals testing positive and negative and regression analysis of factors associated with a positive test demonstrate the importance of ongoing universal testing. As in previous studies, injecting status was the most important factor in HCV infection, and this mediated the relationship between substance type (heroin and crack) and positivity [10]. Within the cohort testing negative, there was a huge population of PWID and there were not substantial differences between the cohorts testing positive and negative, demonstrating the need for ongoing, regular testing in this population. This is particularly important in Blackpool, where, as in other deprived coastal areas, there is a very transient, vulnerable population that is attracted by cheap housing and which represents a potential inward migration of new HCV infection [15].

Amongst individuals testing positive in this analysis, there was a predominance of heroin and crack use. Nationally, and in Blackpool, the profile of drug use has changed in recent years [16]. Substances such as ketamine and other opioids have an increasing profile and were present in services amongst those testing negative. Dependence on opioids, often initially prescribed, that are taken orally has repeatedly been shown to act as a gateway to injecting heroin, and similarly, ketamine use that starts as oral or intra‐nasal often progresses to injecting [17, 18, 19]. This demonstrates that there is an additional population at risk of future HCV infection and is particularly relevant given the very long time period in this study between first drug use and HCV diagnosis, suggesting that drug use escalates over several years before resulting in behaviours favouring HCV transmission.

As the service moves forward, there is also the likelihood of reinfection amongst those previously treated and achieving SVR. The previously mentioned French study showed reinfection rates of 17% during the follow‐up period, whereas a Scottish study showed similarly high reinfection, with poor detection after the initial year post SVR [14, 20]. Both studies emphasise the need for re‐testing and re‐treatment to be offered without stigma or discrimination [14, 20].

This analysis has focused on individuals linked to drug and alcohol services in Blackpool; however, there will inevitably be a large cohort unknown to services and there may have been an element of selection bias whereby the study cohort represents individuals more likely to engage with healthcare services than the wider population at risk of HCV. Indeed, 2023/2024 data suggest that 56.1% of individuals using opiates and/or crack in Blackpool were not accessing specialist drug misuse services, in line with the national figure of 52.9% [21]. Micro‐elimination represents treatment as prevention, whereby treating infected individuals reduces the prevalence of HCV, its onwards transmission and the incidence of new cases [13]. As such, there will be benefit to PWID within Blackpool who do not currently access services. However, future efforts should include additional pathways to engage these individuals. Although there has been success in other local settings, with HMP Kirkham (the male prison serving the Blackpool area) also achieving HCV micro‐elimination, studies from various settings have demonstrated the effectiveness of other models for engaging PWID who do not seek out services [22]. For example, national HCV elimination efforts in Iceland and France have included focused testing in homeless shelters and hostels [11, 14]. This is worthwhile because, although resource‐intensive in terms of testing and treating clients, HCV micro‐elimination efforts are cost‐saving to the healthcare system, with long‐term reductions in disease severity and liver‐related events offsetting initial costs [23].

### 4.4 Limitations

A key limitation of this study was that data were provided by drug and alcohol services only and did not contain information about other HCV risk factors that would have enhanced the analysis, such as previous blood transfusions, prison sentences, geographical origin, sexually transmitted infections, piercings and tattoos. Similarly, additional information on factors such as comorbidities and disability was only available for those individuals testing positive, which limited the regression analysis around predictors of a positive test. As there were small numbers of positive cases, it is also possible that regression analyses may have been under‐powered and, therefore, associated with unstable estimates and wide confidence intervals.

An important limitation of this study, and of micro‐elimination in general, is the distinction between service‐level micro‐elimination criteria and elimination of HCV transmission. Micro‐elimination criteria represent administrative targets for services and are not the same as epidemiological elimination of HCV. Nevertheless, these criteria represent measurable and achievable standards for services to measure and define progress and improvement.

Finally, although data were available in terms of treatment outcomes for those testing positive, it would also have been interesting to understand the extent of liver scarring and cirrhosis, particularly given the long average time since first drug use.

## 5 Conclusion

In this study, we have described a multi‐faceted, multi‐stakeholder approach to achieve HCV micro‐elimination in Blackpool, England, and the associated outcomes. Although successful in the short‐term, ongoing success will depend on sustained culture change within drug and alcohol services to maintain momentum around HCV testing and treatment, including re‐treatment. Drawing people who use drugs into services through existing routes, with the potential for future targeted outreach, is pivotal. This enables individuals to be tested and treated for HCV, as well as provided with core harm reduction approaches that prevent the ongoing transmission of HCV, as well as other drug‐related harm.

## Author Contributions

**Emma L. MacFarlane**: conceptualization, methodology, data curation, investigation, project administration, writing – original draft, writing – review and editing. **Dianne C. Draper**: conceptualization, methodology, investigation, project administration, supervision, writing – original draft, writing – review and editing. **Sarah E. Blagden**: conceptualization, methodology, investigation, formal analysis, software, visualization, writing – original draft, writing – review and editing.

## Funding

The authors have nothing to report.

## Ethics Statement

The authors confirm that this study adheres to the ethical policies of Public Health Challenges. Human participants were not involved in this research study, which utilised secondary data only. The project received ethical approval from Blackpool Council's Research Governance Service. Information governance approval was provided by Blackpool Council's Information Governance Team.

## Conflicts of Interest

The authors declare no conflicts of interest.

## Data Availability

As the project utilised drug and alcohol service data that are not in the public domain, we are not able to make the dataset routinely available. However, we will consider direct requests for data access on a case‐by‐case basis.
